# The Impact of the CTHRSSVVC Peptide Upon Experimental Models of *Trypanosoma cruzi* Infection

**DOI:** 10.3389/fcimb.2022.882555

**Published:** 2022-05-06

**Authors:** Gabriela Rodrigues Leite, Denise da Gama Jaén Batista, Ana Lia Mazzeti, Rosemeire Aparecida Silva, Ademar Benévolo Lugão, Maria de Nazaré Correia Soeiro

**Affiliations:** ^1^ Laboratório de Biologia Celular, Instituto Oswaldo Cruz (FIOCRUZ), Fundação Oswaldo Cruz, Rio de Janeiro, Brazil; ^2^ Universidade do Estado de Minas Gerais (UEMG), Laboratório de Parasitologia Aplicada, Unidade Passos, Belo Horizonte, Brazil; ^3^ Hospital das Clínicas, Faculdade de Medicina, Universidade de São Paulo (HCFMUSP), São Paulo, Brazil; ^4^ Instituto de Pesquisas Nucleares e Energéticas (IPEN), CNEN, São Paulo, Brazil

**Keywords:** Chagas disease, *Trypanosoma cruzi*, experimental chemotherapy, CTHRSSVVC peptide, immunomodulation

## Abstract

Chagas disease (CD), caused by the hemoflagellate protozoan *Trypanosoma cruzi*, affects more than six million people worldwide and presents an unsatisfactory therapy, based on two nitroderivatives, introduced in clinical medicine for decades. The synthetic peptide, with CTHRSSVVC sequence (PepA), mimics the CD163 and TNF-α tripeptide “RSS” motif and binds to atheromatous plaques in carotid biopsies of human patients, spleen tissues, and a low-density lipoprotein receptor knockout (LDLr−/−) mouse model of atherosclerosis. CD163 receptor is present on monocytes, macrophages, and neutrophils, acting as a regulator of acute-phase processes and modulating aspects of the inflammatory response and the establishment of infections. Due to the potential theranostic role of PepA, our aim was to investigate its effect upon *T. cruzi* infection *in vitro* and *in vivo*. PepA and two other peptides with shuffled sequences were assayed upon different binomials of host cell/parasite, including professional [as peritoneal mouse macrophages (PMM)] and non-professional phagocytes [primary cultures of cardiac cells (CM)], under different protocols. Also, their impact was further addressed *in vivo* using a mouse model of acute experimental Chagas disease. Our *in-vitro* findings demonstrate that PepA and PepB (the peptide with random sequence retaining the “RS” sequence) reduced the intracellular parasitism of the PMM but were inactive during the infection of cardiac cells. Another set of *in-vitro* and *in-vivo* studies showed that they do not display a trypanocidal effect on bloodstream trypomastigotes nor exhibit *in-vivo* efficacy when administered after the parasite inoculation. Our data report the *in-vitro* activity of PepA and PepB upon the infection of PMM by *T. cruzi*, possibly triggering the microbicidal arsenal of the host professional phagocytes, capable of controlling parasitic invasion and proliferation.

## Introduction

According to the World Health Organization (WHO), neglected tropical diseases (NTDs) are communicable diseases that lack attention, care, and investments in areas of research and development of vaccines and medicines, affecting very poor communities with impaired public health access to early diagnosis and treatment (WHO, 2022).

Among the list of 20 NTDs, Chagas disease (CD), also known as American trypanosomiasis, affects more than six million people worldwide and is responsible for about 10,000 deaths per year (Sales Junior et al., 2017).

The etiological agent of CD is the protozoan *Trypanosoma cruzi* that presents different forms in both vertebrate and invertebrate hosts and that can be transmitted by distinct pathways, including via a vector insect and the oral route (Chatelain, 2017; Lidani et al., 2019). CD has two phases, acute and chronic, and since its discovery in 1909 by Carlos Chagas, it poses as a serious public health problem (WHO, 2022). Vaccines are not available and then control and treatment are exclusively related to drug therapy based on two very old pharmacological entities—benznidazole (Bz) and nifurtimox (NF)—introduced in clinical use for more than five decades (Soeiro and de Castro, 2009; Soeiro and de Castro, 2011; Soeiro, 2022). Both nitroderivatives have serious drawbacks including their limited efficacy, especially in the later chronic phase, and severe side effects with discontinuation (about 20%) of treatment (Soeiro and de Castro, 2011; Vieira et al., 2019; de Araújo et al., 2020).

Different experimental therapeutic approaches have been pursued aiming to find new drug candidates to face this sad scenario. The search includes several strategies such as i) repositioning studies of drugs already available in the market for other illness conditions, ii) identification of new antiparasitic agents using diverse libraries (composed of natural and synthetic products), iii) design and synthesis of new chemical entities selectively directed to molecular targets as well as iv) combined therapy, and v) molecular hybridization (hybrid compounds) combining two molecules (or parts of them) in a single new chemical entity to act on multiple targets of interest (Scarim et al., 2018; Simões-Silva et al., 2019; Soeiro, 2022).

The knowledge regarding the mechanisms involved in the pathological manifestations of CD has still not been fully gained (Soeiro, 2022). This knowledge could bring relevant contributions to the identification of more selective and complementary therapy for CD. In fact, despite being able to control and reduce the parasitic load in the acute phase, the host immune response and triggered inflammation are not able to fully eliminate the infection, and a progressive unbalanced inflammatory response (triggered by residual parasitism) is associated with the deteriorated conditions and may represent an important target for CD therapy (Benvenuti et al., 2017; Soeiro, 2022).

An interesting study demonstrated the ability of a peptide sequence—CTHRSSVVC (PepA), which mimics the CD163 molecule tripeptide “RSS” motif (Etzerodt et al., 2014) to bind inflammatory foci present in atheroma plaques in carotid biopsies of human patients, spleen tissues, and a low-density lipoprotein receptor knockout (LDLr−/−) mouse model of atherosclerosis (Silva et al., 2016). CD163 is present on professional phagocytes such as monocytes, macrophages, and neutrophils; eliminates hemoglobin and haptoglobin complexes; and acts as an acute-phase controller. Moreover, it plays a role in modulating inflammatory responses and the establishment of infections (Fabriek et al., 2009). *In-vitro* studies showed that the tracer [18F]AlF-NODAMP-C6-CTHRSSVVC binds CD163 receptors allowing a clear visualization of cancer cells using positron emission tomography (PET), although it was not able to detect subtle differences in CD163 levels of tumors induced by different treatments (Fernandes et al., 2021). No selective binding of the sulfo-Cy5-CTHRSSVVC peptide into macrophages could be noticed *in vitro*, but this tracer accumulates in a 4T1 tumor-bearing BALB/c mice model (Kovacs et al., 2022). Besides CD163, the RSS motif is likewise present in TNF-α (Etzerodt et al., 2014; Riethmueller et al., 2016; Zunke and Rose-John, 2017), stimulating its analysis in additional theranostic approaches (Silva et al., 2016; Fernandes et al., 2021; Kovacs et al., 2022).

Thus, as these previous reports suggest the potential theranostic role of PepA, our aim was to investigate its effect upon *T. cruzi* infection *in vitro* and *in vivo*. PepA and PepB (the peptide with random sequence retaining the “RS” sequence) reduced the intracellular parasitism of peritoneal mouse macrophages but were inactive during the infection of cardiac cells. Additional *in-vitro* and *in-vivo* studies showed that they do not display a trypanocidal effect on bloodstream trypomastigotes nor exhibit *in-vivo* efficacy when administered in a mouse model of acute *T. cruzi* infection after the parasite inoculation. The bulk of our data demonstrates the *in-vitro* activity of PepA and PepB upon the infection of PMM by *T. cruzi*, possibly triggering the microbicidal arsenal of the host professional phagocytes, capable of controlling parasitic invasion and proliferation.

## Materials and Methods

### Tested Compounds

**Figure 1:**
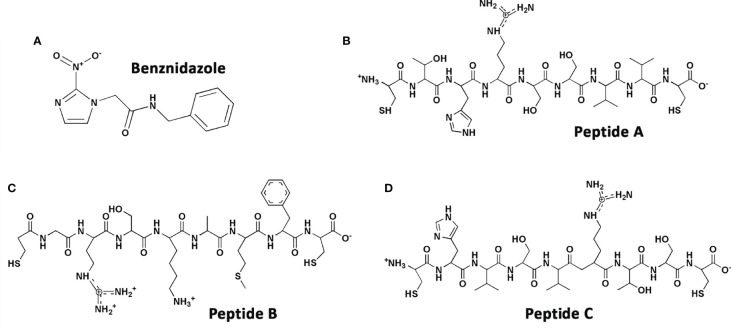
Chemical structures of **(A)** benznidazole, **(B)** PepA, **(C)** PepB, and **(D)** PepC.

Peptide sequences (**Figure 1**) were synthesized by the Chinese Peptides Company (Hangzhou, China) and fully characterized by nuclear magnetic resonance (NMR); carbon, hydrogen, and nitrogen (C, H, N) composition analyses; and MALDI/FAB mass spectrometry as reported (Silva et al., 2016). Stock solutions were prepared at 6 mM in sterile deionized water. The original sequence (CTHRSSVVC) which mimics the “RSS” motif of the CD163 molecule was named peptide A (PepA), the random sequence retaining the “RS” sequence of peptide A was called peptide B (CGRSKAMFC, PepB), and a scrambled sequence of peptide A was named peptide C (CHVSVRTSC, PepC). The reference compound for trypanocidal activity was the nitroderivative benznidazole [N-benzyl-2-(2-nitroimidazol-1-yl) acetamide2-nitroimidazole, Bz] (Laboratório Farmacêutico do Estado de Pernambuco, Brazil) (**Figure 1**), which was prepared in a stock concentration of 50 mM diluted in dimethylsulfoxide (100% DMSO), with the final in-test concentration never exceeding 0.6% for *in-vitro* experiments to avoid non-specific toxicity. For *in-vivo* assays, the peptides were diluted daily and prepared in sterile and deionized water. The reference drug was diluted in a solution composed of sterile and deionized water with 3% of Tween 80 (Guedes-da-Silva et al., 2016).

### Parasites

Bloodstream trypomastigote forms of the Y strain of *T. cruzi* were obtained from male Swiss Webster mice at the peak of parasitemia by cardiac puncture (Meirelles et al., 1986; Batista et al., 2010).

### Mammalian Host Cells

Primary cultures of mouse embryonic cardiac cells (CM) were obtained as described (Meirelles et al., 1986). CM were seeded into 24-well plates with 50 × 10^3^ cells per well. Peritoneal mouse macrophages (PMM) were obtained by peritoneal lavage of male Swiss mice (Santos et al., 2020). PMM were plated into 96-well plates (50 × 10^3^ cells per well) and 24-well plates (30 × 10^4^ cells per well). All cultures were maintained at 37°C/5% CO_2_.

### Mammalian Toxicity

The toxicity of the peptides was investigated upon PMM using the alamarBlue test (Invitrogen, Waltham, MA, USA) according to the manufacturer’s recommendations (Timm et al., 2014; Guedes-da-Silva et al., 2016). PMM were treated for 48 h with increasing concentrations of the tested peptides (up to 500 µM). Following the exposition period, the colorimetric test was performed with alamarBlue^®^ (cell viability detection test) and absorbance was determined by a spectrophotometer (570 and 600 nm). All assays were performed in triplicate in at least two individual assays (Timm et al., 2014; Guedes-da-Silva et al., 2016).

### The Activity of the Peptides on Intracellular Forms of the Y Strain Present in Primary Cell Cultures (PMM and CM)

The effect of the peptides on intracellular forms in PMM was performed under the following protocols: i) *pretreatment*: PMM were rinsed and incubated for 24 h with different concentrations of the tested peptides and with Bz up to a maximum concentration of 50 μM. After incubation, the culture medium was replaced to remove the tested compounds and PMM were infected for 2 h with trypomastigote forms of *T. cruzi* (Y strain, M.O.I. 10 parasites:1 cell). Then, cultures were rinsed to remove the non-internalized parasites and incubated for 48 h at 37°C with RPMI culture medium. ii) *Posttreatment*: PMM were infected for 2 h with *T. cruzi* (Y strain, M.O.I. 10 parasites:1 cell), rinsed, and incubated for 24 h at 37°C. PMM were then incubated for 48 h at 37°C with increasing concentrations (up to 50 μM) of the peptides and Bz.

The effect of the peptides on intracellular forms of *T. cruzi* present in CM was performed as follows: *i) pretreatment*: CM cultures were incubated for 24 h with different concentrations of the peptides and Bz (up to 50 μM). Then, the cultures were rinsed to remove the compounds and infected for 24 h with bloodstream trypomastigote forms of *T. cruzi* (Y strain, M.O.I. 10 parasites:1 cell). After infection, the samples were rinsed to remove non-internalized parasites and incubated for 48 h at 37°C/5% CO_2_. *ii) Posttreatment*: CM were infected with bloodstream trypomastigote forms (Y strain, M.O.I. 10:1), and after 24 h, the cultures were washed to remove non-internalized parasites and incubated for 48 h at 37°C/5% CO_2_ with increasing concentrations of peptides (up to 50 μM).

After the different assays, PMM and CM were rinsed with phosphate-buffered saline (PBS) and fixed with Bouin solution. Then, the samples were stained with Giemsa for quantification (under light microscopy) of the percentage of infected host cells, the number of parasites per cell to calculate the infection index (multiplication between these two factors). Infected cultures not subjected to the treatments were used as negative controls (Guedes da Silva et al., 2016).

All assays were performed in duplicates in at least two individual assays (Timm et al., 2014; Guedes-da-Silva et al., 2016).

### The Activity of the Peptides on Bloodstream Trypomastigote Forms

Bloodstream trypomastigotes (Y strain) were incubated with the tested peptides and Bz up to a concentration of 100 µM. After 2 and 24 h of incubation at 37°C, the number of live parasites (identified by their characteristic morphology and movement) was determined under a light microscope by quantification in a Neubauer chamber to determine the EC_50_ values. Controls were performed with parasites kept under the same conditions, in the absence of peptides (de Araújo et al., 2019).

*In-vitro* studies represent the analysis of two individual assays performed in duplicates.

### Effect of Peptides Upon Experimental Mouse *Trypanosoma cruzi* Infection

Male Swiss Webster mice (18–20 g, 4–5 weeks of age) obtained from the animal facilities of the Institute of Science and Biomodels Technology (ICTB) FIOCRUZ were housed at a maximum of five per cage, kept in a specific-pathogen-free room at 20°C to 24°C under a 12-h light and 12-h dark cycle, and provided sterilized water and chow *ad libitum*. Male mice were used as previous findings showed that they are more vulnerable to experimental infection than females and thus more suitable for therapeutic screenings (Guedes et al., 2015). The animals were acclimated for 7 days before starting the experiments. Infection was performed by intraperitoneal (i.p.) injection of 10^4^ bloodstream trypomastigotes (Y strain). *Trypanosoma cruzi*-infected mice were treated intraperitoneally with 0.1 ml of the tested peptides for up to 10 consecutive days, starting at 5 dpi, which corresponds to (in this experimental model) the parasitemia onset. Only mice with positive parasitemia were used in the infected groups. Benznidazole at 100 mg/kg/day (as the optimal dose) was run in parallel. Peptides were freshly prepared in sterile and distilled water, dosing according to the body weight of the animals. The experimental animal groups were divided as follows: uninfected (uninfected and untreated), untreated (infected and treated with vehicle only), treated with Bz (infected and treated with daily doses of 0.1 ml at 100 mg/kg, by gavage), and treated with peptides (infected and treated with daily doses of 0.1 ml of each peptide at 10 mg/kg). Parasitemia was performed individually by direct counting of the parasite in the blood (5 µl) under a light microscope. Mice were weighed once a week to monitor possible changes in body weight. Mortality was checked daily up to 30 days after treatment and expressed as the percentage of accumulated mortality (% CM) (Ferreira de Almeida Fiuza et al., 2018). *In-vivo* studies were performed twice using five animals per group (*n* = 5).

### Ethics Statement

All procedures were carried out in accordance with the guidelines established by FIOCRUZ Committee of Ethics on Animal Experimentation (CEUA) number L-038/2017.

### Statistical Analysis

The statistical analysis was performed by analysis of variance (ANOVA), the data for the different assays were combined, and the significance was set at a *p-*value of ≤0.05 (Guedes-da-Silva et al., 2016).

## Results

### Cytotoxic Effect of the Peptides on Mammalian Host Cells

Considering the importance of identifying non-toxic agents, the cytotoxic effect of the peptides on PMM was assessed using the alamarBlue test. The findings demonstrated that the tested peptides did not induce loss of cellular viability when PMM were incubated for 48 h (up to 500 µM), while Bz gave LC_50_ value = 333 ± 165 μM (**Table 1**).

**Table 1:** Determination of the cytotoxicity profile (LC_50_ values) of peptides (PepA, PepB, and PepC) and benznidazole (Bz) on the primary culture of peritoneal mouse macrophages (PMM).

	LC_50_ values (mean ± SD/µM)^a^
Bz	333 ± 165
PepA	>500
PepB	>500
PepC	>500

a Analysis represents two individual assays performed in triplicates.

### Effect of the Peptides Upon Infection of PMM by *Trypanosoma cruzi*

**Figure 2:**
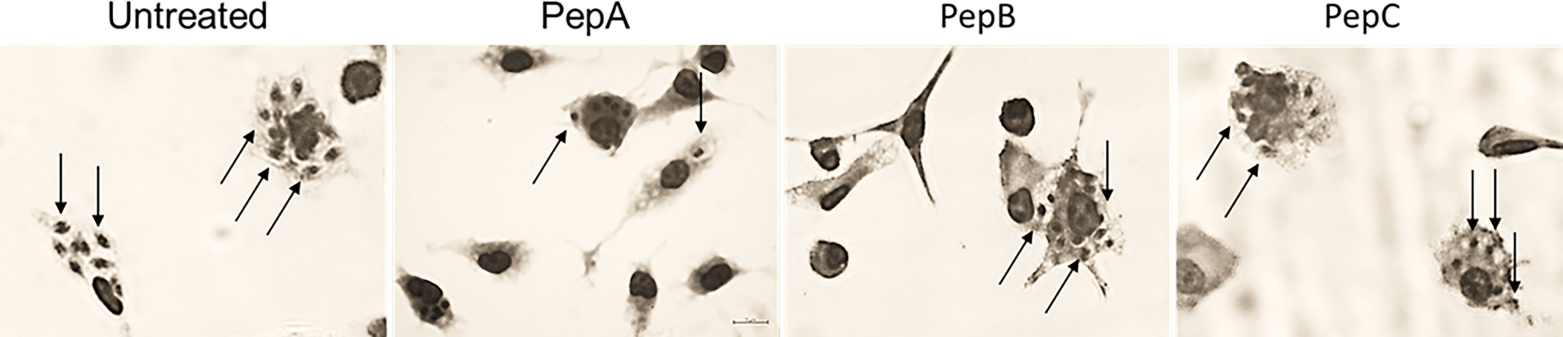
Light microscopy images of peritoneal mouse macrophages pretreated with PepA, PepB, and PepC at 50 µM before infection with *Trypanosoma cruzi*. **(A)** Untreated, **(B)** PepA, **(C)** PepB, and **(D)** PepC. Arrows: intracellular parasites.

The activity of the peptides and Bz upon the infection of PMM with *T. cruzi* [Y strain, discrete typing unit (DTU II)] was evaluated by counting the number of intracellular parasites and the percentage of infected host cells to determine the infection index (II) of Giemsa-stained samples (**Figure 2**). The assays were performed under two different protocols, namely, pre- and posttreatment, by the addition of the tested peptides before and after the establishment of the infection to verify their potential effect directly on the host cells and against the intracellular parasites, respectively (**Tables 2**, **3**). The previous treatment of PMM with PepA and PepB resulted in lower parasite levels as compared with untreated samples, reaching decreases of 62% (*p* = 0.027) and 49% (*p* = 0.119), respectively, while PepC and Bz gave smaller (30% and 22%) and non-significant drops (*p* = 0.684 and *p* = 0.799), respectively (**Tables 2**, **3**). After the establishment of the infection, PepA and PepB induced a non-significant effect on the parasite load, reaching 36% (*p* = 0.169) and 48% (*p* = 0.621) of decreases, respectively, while Bz reached 98% of decline (*p* = 0.066) (**Table 2**).

**Table 2:** Activity of peptide A (PepA), peptide B (PepB), and benznidazole (Bz) on *in-vitro* infection of peritoneal mouse macrophages (PMM) by *Trypanosoma cruzi* (Y strain) submitted to pre- and posttreatment protocols.

	Infection index (mean ± SD) (% reduction)^a^	
	Pre-treatment	Post-treatment
Untreated	109 ± 16	272 ± 178
Bz	ND	6.5 ± 5.9 (98)*
PepA	41 ± 19 (62)**	174 ± 166 (36)^#^
PepB	56 ± 24 (49)^#^	140 ± 108 (48)^#^

Mean ± SD of the infection index (% of reduction).

ND, not determined.

a Analysis of two individual assays performed in duplicates.

*p > 0.066; **p = 0.027; ^#^p > 0.05.

**Table 3:** Activity of peptide C (PepC) and benznidazole (Bz) on *in-vitro* infection of peritoneal mouse macrophages (PMM) by *Trypanosoma cruzi* (Y strain) using pretreatment protocol: mean ± SD of the infection index (% of reduction).

	Infection index (mean ± SD) (% reduction)^a^
Untreated	229 ± 174
PepC	160 ± 113 (30)^#^
Bz	178 ± 176 (22)^#^

a Analysis of two individual assays performed in duplicates.

^#^p > 0.05.

### Effect of the Peptides Upon Infection of CM by *Trypanosoma cruzi*

The activity of PepA and PepB was additionally investigated using primary cultures of mouse cardiac cells infected with the Y strain. As depicted in **Table 4**, both peptides only caused modest and non-significant (*p* > 0.05) reductions in parasitism of CM, reaching maximum values of 35% inhibition of infection rates at the highest concentration (50 μM), while Bz resulted in 96% of decline (**Table 4**).

**Table 4:** Activity of peptides A and B and benznidazole (Bz) on *in-vitro* infection of cardiac cell cultures by *Trypanosoma cruzi* (Y strain) using pre- and posttreatment with peptides (50 µM) and Bz (5.5 µM) used as a reference drug.

	Infection index (mean ± SD) (% reduction)^a^	
	Pre-treatment	Post-treatment
Untreated	378 ± 113	394 ± 162
Bz	386 ± 56 (0)^#^	14.57 ± 7 (96)*
PepA	288 ± 112 (24)^#^	320.8 ± 154 (19)^#^
PepB	325 ± 86 (14)^#^	253.9 ± 141 (35)^#^

Reduction of the CM infection by T. cruzi: mean ± SD of the infection index (% of reduction).

a Analysis of two individual assays performed in duplicates.

*p = 0.0107; ^#^p > 0.05.

### Effect of the Peptides on Bloodstream Forms of *Trypanosoma cruzi*

To check the potential direct effect of the peptides upon the other relevant form of *T. cruzi* to mammalian infections, the activity of the peptides was assayed upon extracellular bloodstream trypomastigotes (Y strain) through light microscopy. The findings demonstrated that all peptides were inactive against bloodstream forms (EC_50_ > 100 µM), while Bz achieved likely potency (EC_50_ value = 12 µM) (**Table 5**).

**Table 5:** The biological effect of the peptides and benznidazole (EC_50_ values at µM) against bloodstream trypomastigotes of *Trypanosoma cruzi* (Y strain) after 2 and 24 h of incubation at 37°C.

EC_50_ values (mean ± SD*/*µM)^a^		
	2 h	24 h
Bz	–	12
PepA	>100	>100
PepB	>100	>100
PepC	>100	>100

a Analysis of two individual assays performed in duplicates.

### Effect of the Peptides *In Vivo*

**Figure 3:**
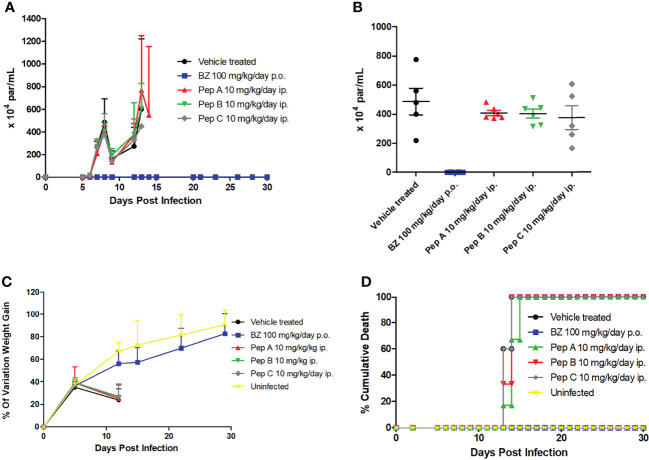
Effect of peptides and benznidazole upon a mouse model of acute *Trypanosoma cruzi* infection. **(A)** Parasitemia curve, **(B)** data dispersion of blood parasitism at 8 days postinfection, **(C)** ponderal curve, and **(D)** cumulative mortality.

Finally, *in-vivo* assays were conducted to explore the potential therapeutic effect of the peptides upon a mouse model of acute *T. cruzi* infection (Swiss mice infected with the Y strain), under administration for 5–10 consecutive days with 10 mg/kg/day, *via* the intraperitoneal route, starting at the parasitemia onset (5 dpi). Our findings (**Figures 3A, B**) show that all peptides displayed a maximum of 23% of parasitemia decline (8 dpi), while Bz (optimal dose of 100 mg/kg/day) fully suppressed (>99%) the parasitemia at 8 dpi, corresponding to the parasitemia peak (**Figure 3B**). Also, the ponderal curve demonstrates that all peptides were unable to protect against weight loss induced by *T. cruzi* infection, which was clearly reverted during Bz administration (**Figure 3C**). Indeed, only the reference drug gave 100% of animal survival, while all peptides reached 100% of cumulative death as soon as at 15 dpi, similar to the vehicle-treated mice group (**Figure 3D**).

## Discussion

The peptide with CTHRSSVVC sequence (PepA) mimics the CD163 and TNF-α tripeptide “RSS” motif. The CD163 receptor is present on monocytes, macrophages, and neutrophils, acting as a regulator of acute-phase processes and modulating aspects of the inflammatory response and the establishment of infections (Etzerodt et al., 2014; Silva et al., 2016). Hence, due to previous reports regarding the theranostic role of PepA, our aim was to evaluate its effect upon *T. cruzi* experimental infection *in vitro* and *in vivo.* Besides PepA, two other sequences were assessed: one that maintains the “RS” region (PepB) and another that maintains all the amino acids in scrambled order to the original sequence (PepC). As CD163 is present in professional phagocytes, these peptides were screened in PMM by means of distinct protocols exploring the potential cytotoxic profile on mammalian cells as well as their effect on *T. cruzi* infection before (pretreatment) and after parasite interaction (posttreatment). Our data showed that all peptides showed a non-toxic profile, which is a desirable characteristic for new drug candidates (Gupta et al., 2015).

PepA and PepB reduced the infection indexes of *T. cruzi*-infected PMM reaching a statistically significant (*p* = 0.027) maximum decline of 62% when the CTHRSSVVC sequence was added prior to parasite interaction. Accordingly, PepC, the one with scrambled sequence, only reached a non-significant decrease of 30% when added prior to protozoan infection, arguing in favor of the specificity of the RS motif present in PepA and PepB, possibly triggering a microbicidal response induced by the RS sequence region. On the other hand, the lack of activity of the peptides on the trypomastigote forms suggests that the effect of PepA and PepB is on PMM metabolism rather than on the parasite itself due to their inability to lyse bloodstream forms.

No major effect was noticed when cardiac cell cultures were used as host cells, supporting the hypothesis that the sequence region “RS” of PepA and PepB may selectively activate professional phagocytes such as PMM by an immunomodulatory pathway. The lack of therapeutic activity against bloodstream trypomastigotes was also corroborated by *in-vivo* approaches demonstrating only a mild reduction of the parasitemia in mice treated with the peptides after *T. cruzi* infection. Although the “RSS” motif is a conserved sequence present only in human and monkey CD163, its soluble form (sCD163) is likewise detectable in mouse serum and is present in TNF-α (Etzerodt et al., 2014; Riethmueller et al., 2016; Zunke and Rose-John, 2017). Macrophages are notoriously heterogeneous. They can adopt a spectrum of phenotypes from the anti-inflammatory M2-type state (CD163) to the pro-inflammatory M1-type state (TNF-α). However, the inflammatory phenotype of the M1 versus M2 macrophage is not constant, which probably reflects the plasticity of monocyte-derived cells in response to microenvironmental changes (Sica et al., 2006; Moghaddam et al., 2018). In this context, their potential biological effect may depend on how the peptide will be presented for specific binding to occur and where they will bind. Since *T. cruzi* infection stimulates the activation of both M1 and M2 macrophages (Zanluqui et al., 2015), further studies will be necessary to evaluate the mode of action of PepA and PepB on *T. cruzi* experimental infection. In fact, in addition to CD163, the RSS motif is present in TNF-α and a soluble CD163 exists in mice, and further studies are necessary to reveal the mode of action of the peptides. These studies would include measurements of different inflammatory mediators *in vitro* besides evaluating the response upon the infection of M1 and M2 macrophage populations as well as the use of distinct *in-vivo* models and regimens (e.g., pretreatment of mice with peptide before parasite inoculation of murine models of acute and chronic infection). Moreover, other factors must be considered during the experimental assays such as peptide stability *in vivo*, levels of macrophage subpopulations, and different types and kinetics of inflammation in the acute and chronic models of *T. cruzi* infection (Silva et al., 2016; Fernandes et al., 2021; Kovacs et al., 2022).

The bulk of our findings suggests that the non-toxic profile of PepA and PepB along with their ability to diminish PMM parasitism *in vitro* justifies additional studies aiming to open new perspectives for alternative therapies for CD.

## Data Availability Statement

The raw data supporting the conclusions of this article will be made available by the authors, without undue reservation.

## Ethics Statement

All procedures were carried out in accordance with the guidelines established by FIOCRUZ Committee of Ethics on Animal Experimentation (CEUA) number L-038/2017.

## Author Contributions

GL contributed to the *in-vitro* and *in-vivo* assays and wrote and edited the entire manuscript. DB contributed to the *in-vivo* assays and analyzed the data. AM contributed to the *in-vivo* assays. RS provided the peptides and reviewed the manuscript. AL provided the peptides and reviewed the manuscript. MS was responsible for the project and methodology design, analyzed the data, and edited and reviewed the manuscript. All authors contributed to the article and approved the submitted version.

## Funding

The Carlos Chagas Filho Foundation for Research Support of the State of Rio de Janeiro (FAPERJ), National Council for Scientific and Technological Development (CNPq), Oswaldo Cruz Foundation, PAEF/CNPq/FIOCRUZ, PDTIS, and CAPES funded this study. MS is a research fellow from CNPq and CNE.

## Conflict of Interest

The authors declare that the research was conducted in the absence of any commercial or financial relationships that could be construed as a potential conflict of interest.

## Publisher’s Note

All claims expressed in this article are solely those of the authors and do not necessarily represent those of their affiliated organizations, or those of the publisher, the editors and the reviewers. Any product that may be evaluated in this article, or claim that may be made by its manufacturer, is not guaranteed or endorsed by the publisher.
